# Low energy availability, the gut microbiome, and bone health in athletes: a mechanistic narrative review based on athlete evidence and clinical analogues

**DOI:** 10.3389/fnut.2026.1901299

**Published:** 2026-07-20

**Authors:** Adam Wagner, Aino Kuljukka, Michal Kumstat, Johanna K. Ihalainen

**Affiliations:** 1 Department of Sport Performance and Exercise Testing, Faculty of Sports Studies, Masaryk University, Brno, Czechia; 2 Faculty of Sport and Health Sciences, University of Jyvaskyla, Jyvaskyla, Finland

**Keywords:** athletes, bone health, endocrine function, gut microbiome, intestinal permeability, low energy availability, relative energy deficiency in sport, short-chain fatty acids

## Abstract

Low energy availability (LEA) is a central aetiological factor in Relative Energy Deficiency in Sport (REDs) and is frequently associated with impaired skeletal health in athletic populations, although skeletal responses can be heterogeneous. However, athletes with apparently similar energetic and training exposures can differ in bone mineral density, bone turnover and bone stress injury risk, indicating that additional physiological mediators may modify the skeletal response to under-fuelling. The gut microbiome has emerged as a plausible candidate because microbial metabolites, intestinal barrier integrity, immune signalling and endocrine pathways can influence bone remodelling. Direct studies integrating energy availability, gut microbiome profiling and bone outcomes in athletes are currently lacking. This narrative review therefore synthesises athlete evidence for the LEA-bone relationship and uses clinical and preclinical analogues of chronic energy deficiency to develop a testable gut-bone framework for sport. The accumulated evidence from athletes primarily supports the direct LEA-bone relationship, whereas the candidate gut-bone microbiome component remains a biologically plausible hypothesis based on clinical and preclinical models. Specifically, evidence from athletes supports LEA and REDs risk as contributors to lower bone mineral density, altered bone microarchitecture, suppressed bone formation markers and bone stress injury risk, although findings vary by sex, sport type, skeletal loading, assessment method and timing. Evidence from anorexia nervosa and other undernutrition models suggests that energy deficiency can be accompanied by altered microbial diversity, depletion of short-chain fatty acid-producing taxa, lower short-chain fatty acid availability, impaired barrier function and low-grade inflammation. Mechanistically, short-chain fatty acids, endotoxin-mediated inflammation, insulin-osteocalcin signalling, bile acid pathways and amino acid metabolites may intersect with canonical REDs endocrine disturbances to influence bone remodelling. The available evidence does not establish a causal gut-mediated pathway in athletes, but it supports a biologically plausible model that should be tested in prospective athlete cohorts using integrated assessments of energy availability, diet, training load, microbiome composition and function, endocrine status, bone turnover and bone structure.

## 1 Introduction

Low energy availability (LEA) occurs when dietary energy intake is insufficient to support the combined energetic demands of exercise and basic physiological function after accounting for fat-free mass. When LEA is sustained or clinically consequential, it can contribute to Relative Energy Deficiency in Sport (REDs), a broader syndrome characterised by impaired health, performance and adaptation across multiple physiological systems (1, 2). Bone health is one of the most clinically important outcomes within this framework. Evidence from experimental and observational work indicates that LEA can suppress osteoanabolic signalling, disrupt reproductive and metabolic hormones, alter bone turnover and increase the risk of low bone mineral density (BMD), impaired microarchitecture and bone stress injury in both women and men (3–9).

Despite this well-established relationship, the skeletal response to LEA is not uniform. Athletes differ in sport type, mechanical loading history, diet quality, fibre and protein intake, body composition, maturation, menstrual status, sex steroid exposure, endocrine resilience and the accuracy with which energy availability can be measured in free-living conditions. Bone outcomes are also temporally complex: current energy availability may change over days, bone turnover markers may respond over days to weeks, whereas BMD reflects cumulative exposure over months to years. These features help explain why some studies report apparently anomalous findings, such as lower BMD in athletes classified as higher energy availability or no clear association between calculated energy availability and bone outcomes (10–16). They also highlight the need to consider additional physiological mediators (e.g., inflammatory pathways, microbial metabolites, intestinal barrier function, and endocrine signalling) that may modify skeletal adaptation to under-fuelling.

The gut microbiome has become a plausible candidate in this context. Athlete cohorts show gut microbial features that differ from non-athlete populations, and these features appear to vary with habitual diet, training load, sport type and cardiorespiratory fitness (17–19). Beyond sport, a growing gut-bone literature suggests that microbial metabolites, intestinal barrier function and immune tone can influence bone turnover (20–23, 105). Short-chain fatty acids (SCFAs), especially acetate, propionate and butyrate, are particularly relevant because they are produced by bacterial fermentation of non-digestible carbohydrates and can support epithelial barrier function, regulate inflammation and influence osteoclast and osteoblast activity (19, 22, 24). Detailed characteristics of human gut microbiome-bone studies in clinical and general populations are provided in Supplementary Table S4.

However, the central limitation is clear: no original athlete study has yet directly tested whether objectively or clinically defined LEA is associated with gut microbiome composition, microbial metabolites and bone outcomes within the same cohort. As a result, the field currently depends on triangulation. Athlete studies establish the LEA-bone problem; sport microbiome studies show that athletic training and diet relate to gut microbial ecology; clinical undernutrition models, especially anorexia nervosa, provide evidence that chronic energy deficiency can alter the gut microbiota (25–35); and gut-bone studies in clinical and preclinical populations provide mechanistic candidates. The purpose of this review is therefore not to claim that a gut-mediated mechanism has been demonstrated in athletes, but to define a testable physiological framework.

The aim of this narrative review is to synthesise evidence linking LEA and bone health in athletes and to evaluate whether gut microbial pathways could plausibly contribute to variability in skeletal outcomes. To avoid over-extrapolation, we distinguish LEA and REDs in sport from broader chronic energy deficiency and explicitly treat clinical populations as analogues for hypothesis generation rather than direct substitutes for athletic cohorts.

## 2 Search approach and scope

This article is a narrative review informed by a structured literature search. The original search strategy was conducted in PubMed, Web of Science and Scopus through February 2025 and targeted three evidence areas: energy availability and bone health, energy availability and gut microbiota, and gut microbiota and bone health. Human studies published in English were considered for the evidence synthesis. Animal, *in vitro* and review articles were not included in the primary study tables, although selected mechanistic and review sources were used to interpret physiological pathways. The original search identified 86 studies for synthesis, including five additional studies identified through citation tracking.

For this revision, targeted literature searches were also performed to address specific mechanistic gaps raised during peer review, including insulin-osteocalcin signalling, oligosaccharide/prebiotic pathways, amino acid metabolites and spaceflight-related gut-bone models. These targeted additions are used only to strengthen mechanistic interpretation and future research recommendations. Because the present article is framed as a narrative Review rather than a Systematic Review, no formal meta-analysis or risk-of-bias scoring was performed. This choice reflects the heterogeneity of the evidence base, the absence of direct athlete LEA-microbiome-bone studies and the primarily hypothesis-generating purpose of the article. This narrative approach is consistent with established typologies of review articles (85).

To ensure a rigorous and balanced interpretation, we evaluate the literature using four distinct levels of evidence. This structured grading is essential because the biological plausibility of a gut-bone pathway in preclinical models does not by itself establish clinical relevance in athletic populations. The first and highest level of evidence (Level 1) is assigned to athlete or physically active cohorts in which energy availability, REDs risk, bone turnover, BMD, microarchitecture or bone stress injury outcomes were measured. A second level of evidence (Level 2) includes athlete microbiome studies that inform how training and diet shape gut ecology, even when LEA was not measured. A third level of evidence (Level 3) includes clinical undernutrition and eating-disorder cohorts that provide analogues of chronic energy deficiency. A fourth level of evidence (Level 4) includes mechanistic gut-bone evidence from osteoporosis, ageing, animal and spaceflight models. Table 1 provides a comprehensive summary and definition of the key energetic and clinical constructs used throughout this review.

**Table 1:** Terminology and key constructs used in energy availability and sport-specific bone health literature.

Construct/Term	Definition and physiological meaning	Application and measurement in sport
Energy availability (EA)	Dietary energy intake minus exercise energy expenditure, normalised to fat-free mass (FFM). Represents the energy remaining for cellular and physiological functions.	Calculated as: (Energy Intake—Exercise Energy Expenditure)/FFM. Expressed in kcal/kg FFM/day.
Low energy availability (LEA)	A metabolic state where EA is insufficient to support physiological health and normal bodily functions.	Historically defined by a threshold of <30 kcal/kg FFM/day in females, though modern models view it as a continuous, threshold-free gradient.
Adaptable LEA	A transient, mild, or acute energy deficit that the body can adapt to without experiencing significant clinical or health impairments.	Typically occurs during short-term training blocks or weight-management phases; represents an evolutionary energy-conservation response.
Problematic LEA	A chronic, severe, or prolonged state of energy deficiency where physiological systems begin to fail or experience major clinical dysfunction.	The primary clinical driver of REDs; results in long-term bone degradation, endocrine suppression, and impaired health/performance.
Relative energy deficiency in sport (REDs)	The comprehensive clinical syndrome driven by problematic LEA. Characterised by physiological function across multiple systems and decrements in performance.	Assessed using clinical frameworks like the IOC REDs Clinical Assessment Tool version 2 (REDs CAT2), which integrates multiple symptoms.
Menstrual dysfunction	A major clinical marker of reproductive energy saving and HPG axis suppression in female athletes (e.g., hypothalamic amenorrhea, oligomenorrhea).	Monitored via menstrual history, cycle tracking, and sex hormone panels (luteinizing hormone, oestrogen).
Resting metabolic rate (RMR) ratio	The ratio of measured resting metabolic rate to predicted resting metabolic rate. Used as an objective, cumulative marker of metabolic suppression.	Measured via indirect calorimetry and compared to predictive equations (e.g., Harris-Benedict). An RMR ratio <0.90 indicates metabolic conservation.
Screening questionnaires	Standardised, symptom-based tools used to identify athletes at risk of LEA or REDs based on clinical signs.	Examples include the Low Energy Availability in Females Questionnaire (LEAF-Q) and the Sport-Specific Energy Availability Questionnaire (SEAQ).

## 3 Low energy availability and bone health in athletes

Bone is one of the best documented physiological systems affected by LEA. Early work focused primarily on female athletes with menstrual disturbances within the Female Athlete Triad model, whereas more recent literature has broadened the framework to include male athletes, REDs risk stratification, endocrine markers, bone turnover and bone stress injury risk (1–3, 36, 37). Across this literature, LEA is most consistently associated with reduced bone formation, impaired bone accrual or lower BMD when under-fuelling is prolonged, clinically meaningful or accompanied by reproductive and metabolic endocrine disruption.

### 3.1 Bone mineral density and bone quality

Most athlete studies support an association between LEA-related indicators and poorer skeletal outcomes, although the strength and anatomical distribution of findings vary. Reductions in BMD are most frequently reported at clinically key sites such as the lumbar spine and femoral neck, as these regions are amongst the most studied, contain a high proportion of metabolically active trabecular bone, and are particularly responsive to alterations in systemic bone metabolism and architecture (4–6, 38–47). Heikura et al. reported a graded decline in BMD across IOC REDs Clinical Assessment Tool version 2 (CAT2) risk categories in a large elite athlete cohort (39). Other studies using tools such as the Low Energy Availability in Females Questionnaire (LEAF-Q), sport-specific clinical interviews or calculated energy availability have identified lower BMD or unfavourable bone outcomes in at-risk athletes, particularly in endurance or weight-sensitive sports (40–45, 47). Table 2 provides a condensed summary of the Level 1 athlete evidence linking LEA, REDs risk, and skeletal outcomes. Detailed characteristics of athlete BMD and bone-quality studies are provided in Supplementary Table S1.

**Table 2:** Condensed interpretation of athlete evidence linking low energy availability (LEA), REDs risk and skeletal outcomes.

Evidence domain	Typical finding	Key interpretation	Main caveats
BMD and bone quality	LEA indicators or higher REDs risk are often associated with lower BMD at the lumbar spine, femoral neck, hip or total body.	Sustained under-fuelling can compromise skeletal accrual and maintenance, especially when combined with reproductive or metabolic endocrine disruption.	BMD reflects long-term exposure; current EA may not match skeletal history; site-specific loading modifies findings.
Bone turnover markers	Short-term LEA commonly suppresses formation markers such as P1NP, osteocalcin or PICP; resorption markers may increase in some contexts.	Bone formation appears particularly sensitive to energetic stress; resorption responses may depend on sex hormones, severity and duration.	Markers are affected by circadian rhythm, feeding, exercise, menstrual status and sampling timing.
Bone stress injury risk	Triad or REDs risk factors, menstrual dysfunction, low BMD and prior injury are associated with greater BSI risk.	Clinical risk is multidimensional and should not be inferred from a single EA calculation.	Prospective injury surveillance is limited; definitions of REDs risk and BSI vary across studies.
Apparently anomalous results	Some studies report null associations or higher BMD in lower-EA groups.	Findings may reflect mechanical loading, sport type, reporting error, timing mismatch or classification differences rather than absence of LEA effects.	Calculated EA, questionnaires and symptom-based tools capture related but non-identical constructs.

However, the literature is not uniform. Several studies reported no association between LEA-related measures and BMD, and a smaller group reported apparently paradoxical findings, such as higher BMD in athletes classified as lower energy availability or lower BMD in athletes with higher calculated energy availability (10–16). These discrepancies should not be interpreted as evidence that LEA is irrelevant to bone. Instead, they point to methodological and biological constraints. First, BMD is a long-term outcome and may not align with a short measurement window for energy intake and expenditure. Second, self-reported dietary intake and exercise expenditure are vulnerable to substantial measurement error, especially in free-living athletes (48). Third, different tools capture different constructs: calculated energy availability, LEAF-Q, SEAQ-I, menstrual status and REDs CAT categories are related but not interchangeable. Fourth, skeletal loading can modify BMD independently of energy availability. Cyclists may have high energy intake but low site-specific bone loading, whereas runners, jumpers, resistance-trained athletes and multidirectional field-sport athletes may receive more osteogenic stimuli despite intermittent under-fuelling.

The sport-type issue is particularly important. Mechanical loading can partly protect bone at loaded sites, whilst non-weight-bearing or low-impact endurance training may leave the skeleton vulnerable even when energy intake appears adequate. This helps explain findings in cyclists, such as those by Chen et al. (15), where competitive cyclists exhibited high energy availability but low BMD. This apparently paradoxical finding can be explained by several factors. First, cycling is a non-weight-bearing sport that lacks the impact-induced skeletal loading necessary to stimulate osteoanabolism. Mechanistically, the absence of high-magnitude mechanical strain reduces the activation of the Wnt/*β*-catenin signalling pathway in osteocytes, which is a primary driver of osteoblast differentiation and bone formation; consequently, even with sufficient energy availability, the skeleton lacks the local mechanical cues required to maintain bone mass. Second, competitive cyclists often face high training volumes and systemic physiological stress (e.g., overtraining), which can elevate cortisol and other catabolic hormones that further suppress bone remodelling. Third, potential interactions with ergogenic aids or weight-management practises (e.g., rapid weight-loss phases not captured by standard energy availability windows) may confound the relationship. Conversely, athletes in impact or combat sports may show preserved or higher BMD despite weight cycling or dietary restriction because repeated high-impact loading stimulates bone formation (16). These interactions argue for interpreting LEA-bone studies through both metabolic and mechanical lenses. Recognising this vulnerability, bone health has become an increasing focus in athletes participating in low-impact or non-weight-bearing sports (e.g., cycling and swimming), many of whom now incorporate targeted osteogenic activities—such as resistance training, plyometrics, and progressive impact-loading exercises—to support bone health and mitigate systemic risk (49). The inclusion of these cross-training modalities is a critical variable to consider when interpreting bone outcomes across sporting populations, as it can modify site-specific skeletal responses even under conditions of energetic stress.

### 3.2 Bone turnover markers

Bone turnover markers provide a more dynamic window into the skeletal response to LEA than BMD. Experimental studies show that short-term LEA can reduce bone formation markers such as procollagen type I N-terminal propeptide (P1NP), osteocalcin or procollagen type I C-terminal propeptide, and in some cases increase resorption markers such as beta-isomer of C-terminal telopeptide of type I collagen (beta-CTX) or urinary C-terminal telopeptide (4, 7, 9, 50–53). Papageorgiou et al. reported reduced P1NP and increased beta-CTX after several days of LEA in women, whereas responses in men were less consistent (9). Ihle and Loucks demonstrated dose-dependent reductions in bone formation markers across progressively lower energy availability conditions (7). More recent short-term interventions in men have shown mixed effects, with some studies reporting changes in endocrine or fatigue markers without clear bone turnover changes (54, 55). Detailed characteristics of bone-turnover studies are provided in Supplementary Table S2.

This heterogeneity likely reflects differences in duration, severity and mode of LEA; sex; hormonal status; feeding pattern; exercise mode; and sampling timing. Bone turnover markers exhibit circadian rhythm and are sensitive to recent feeding, exercise and menstrual status, which can obscure true effects when protocols are not standardised. Nonetheless, the overall pattern is biologically coherent: severe or sustained LEA tends to suppress bone formation, and resorption may increase when LEA co-occurs with hypoestrogenism, low testosterone, high cortisol or inflammatory stimuli.

### 3.3 Bone stress injuries and clinical consequences

The clinical endpoint of greatest concern is bone stress injury. Prospective and cross-sectional evidence indicates that Triad or REDs risk factors, menstrual dysfunction, low BMD, low BMI, prior fracture and restrictive eating behaviours are associated with increased risk of bone stress injuries in female and male athletes (6, 56, 57). Heikura et al. reported that low energy availability was difficult to measure directly but that associated outcomes had substantial impact on bone injury rates in elite distance athletes (6). The clinical implication is that LEA should not be evaluated solely through a single energy availability calculation. Instead, risk assessment should integrate dietary, endocrine, menstrual, injury, performance and bone outcomes.

This broader approach is also relevant for the microbiome hypothesis. If gut-derived signals influence bone remodelling, they are likely to operate alongside, rather than separately from, established REDs pathways. The question is not whether the gut microbiome replaces endocrine mechanisms, but whether microbial function helps explain why similar energetic stress produces different skeletal outcomes across athletes.

## 4 Why the gut microbiome is a candidate link

Exercise and athletic training are associated with differences in gut microbial composition and function, although causality and directionality remain difficult to establish. Athlete cohorts show gut microbial features that differ from non-athlete populations, and these features appear to vary with habitual diet, training load, cardiorespiratory fitness, and the type of athlete (i.e., power athletes, hybrid sport athletes, or endurance athletes) (17–19). For example, endurance athletes (typically consuming high-carbohydrate, high-fibre diets) often show a higher abundance of taxa involved in lactate clearance (e.g., Veillonella) and short-chain fatty acid (SCFA) production (17, 19). Conversely, power and hybrid sport athletes exhibit distinct microbial profiles—often characterised by a higher abundance of protein-fermenting, bacteroidetes-rich, or bile-tolerant taxa—shaped by high-protein dietary patterns and training-related adaptations (18). This matters for REDs because athletes at risk of LEA may simultaneously change multiple microbial drivers: total energy intake, carbohydrate availability, fibre intake, protein distribution, training load, gastrointestinal symptoms, stress, menstrual status and supplement use.

The most important evidence gap is the absence of studies that directly measure LEA and gut microbiome outcomes in athletes. Existing athlete microbiome studies typically characterise training status, performance level or dietary patterns rather than clinically defined LEA. Conversely, LEA and REDs studies typically assess bone, endocrine and performance outcomes but not the gut microbiome. As a result, the proposed LEA-microbiome-bone pathway is currently inferential.

This gap shapes how the review is framed. Athlete data justify the clinical importance of the problem; clinical analogues justify mechanistic hypotheses; but neither can prove that gut microbial changes mediate bone loss in athletes. A cautious interpretation is that the gut microbiome may act as a modifier of skeletal risk under LEA by influencing substrate availability, SCFA production, barrier integrity, inflammatory tone and endocrine signalling. This possibility is sufficiently plausible to guide future studies, but not yet mature enough to guide athlete treatment decisions beyond established REDs care.

## 5 Clinical analogues of chronic energy deficiency

Because direct athlete data are lacking, anorexia nervosa and related undernutrition states provide the most frequently cited human analogues of chronic energy deficiency (96). These populations are not equivalent to athletes with exercise-induced LEA. Anorexia nervosa involves psychiatric, behavioural and gastrointestinal features that differ from inadvertent under-fuelling or sport-driven energy mismatch. Osteoporosis and postmenopausal bone loss likewise involve age, sex-steroid and inflammatory contexts that cannot be directly mapped onto athletes. These differences must be stated clearly to avoid the overgeneralisation criticised in peer review.

Nevertheless, clinical analogues remain useful for hypothesis generation because they share several physiological features relevant to REDs: low energy availability, reduced substrate intake, altered gut motility, low insulin-like growth factor-1 (IGF-1), suppressed hypothalamic–pituitary-gonadal signalling, low triiodothyronine, altered leptin and insulin, stress-axis activation and impaired bone formation (1, 25, 36, 37, 86, 89–95). These overlapping features provide a rationale for asking whether gut microbial responses to undernutrition could interact with skeletal regulation in athletes.

Studies in anorexia nervosa have reported altered gut microbial diversity, shifts in taxonomic composition, depletion of several SCFA-producing taxa and reduced faecal or plasma SCFA concentrations (25–34). Reported taxa vary across cohorts, but reductions in genera such as Roseburia, Faecalibacterium, Ruminococcus and other butyrate-producing organisms appear in multiple studies (26, 28, 32, 33, 58, 59, 106). Broader gut-bone evidence reports context-dependent associations involving Bacteroidetes/Bacteroides, Prevotella, Proteobacteria and Akkermansia; however, directions differ across populations and these taxa should not be treated as athlete biomarkers (109–114, 116, 117). Several studies also suggest that microbial alterations may not fully normalise after short-term weight restoration, implying that gut ecology may lag behind nutritional rehabilitation (26, 28, 30, 31, 60, 61, 108). These findings are relevant to athletes because recovery from REDs may similarly involve a mismatch between apparent energy restoration and slower normalisation of endocrine, skeletal and potentially microbial systems. Detailed characteristics of clinical undernutrition and anorexia nervosa microbiome studies are provided in Supplementary Table S3.

At the same time, the clinical analogue evidence has clear limitations, and the anorexia nervosa literature must be framed cautiously. Although anorexia nervosa provides a useful model of chronic undernutrition, it differs substantially from athlete LEA because of psychiatric factors, illness duration, severity of restriction, gastrointestinal symptoms, constipation, medication exposure, refeeding protocols, and possible laxative use. Laxative use is particularly relevant because it can independently affect gastrointestinal transit, microbial composition, metabolite availability, and intestinal function. Moreover, athletes with LEA are simultaneously exposed to structured exercise, which may independently modify the gut microbiome and, more importantly, bone mineral density through sport-specific mechanical loading. These differences limit the direct translational value of anorexia nervosa microbiome findings for athletic populations. In addition, many clinical studies are cross-sectional and use 16S rRNA sequencing, which provides limited functional resolution. Inflammatory findings in anorexia nervosa are heterogeneous across longitudinal cohorts and meta-analyses (101, 103, 104). Transfer of anorexia nervosa-derived gut microbiota impaired weight gain and behavioural performance in female mice, providing preclinical support for biological activity but not direct evidence in athletes (102). SCFA availability may reflect both microbial capacity and substrate supply. Finally, renourishment studies show that weight gain does not always restore gut microbial composition or function to control levels, indicating that long-term undernutrition can produce persistent ecological changes (26, 27, 29, 30).

An important consideration when translating these clinical findings to sport is the duration and severity of energy deficiency, which dictates whether an athlete experiences “adaptable LEA” or “problematic LEA.” Adaptable LEA represents a transient or mild energy mismatch where the body successfully adapts to lower energy availability without major physiological or clinical impairments, whereas problematic LEA involves prolonged or severe energy deficiency that leads to the clinical syndrome of REDs (2). Whilst anorexia nervosa represents an extreme, chronic, and severe state of problematic LEA, many athletes experience transient, mild, or inadvertent energy deficiencies (e.g., during high-intensity training blocks or weight-sensitive competition phases). The time course of gut microbiome adaptation to energy deficiency remains uncertain. In non-obese adults, a three-week caloric-restriction intervention produced substantial metabolic changes but only limited overall changes in gut microbial composition, illustrating that host and microbial responses may occur on different timescales (97). These early microbial changes might contribute to initial gastrointestinal or metabolic adaptations. However, severe skeletal impairments and profound endocrine suppression (problematic LEA) are more likely to emerge as a consequence of prolonged energy deficiency, where persistent alterations in the gut-bone axis reinforce systemic catabolic pathways. Future prospective studies must characterise this dose–response relationship and the temporal dynamics of gut-bone axis adaptation in athletic populations.

## 6 Mechanistic pathways linking gut function to bone under LEA

To assist in navigating these pathways, proposed mechanisms can be prioritised into primary and secondary candidate pathways based on the current maturity of evidence. The primary, most mature pathways—supported by human clinical data and extensive animal models—are short-chain fatty acids (SCFAs) and gut barrier integrity/endotoxemia. Secondary, more exploratory pathways include bile acid metabolism, amino acid metabolites, and insulin-osteocalcin signalling. These latter mechanisms are supported primarily by preclinical models or metabolomic associations and require direct validation in athletic cohorts. Table 3 provides a summary of these candidate gut-related mechanisms, including their proposed pathways, evidence sources, and Level of Evidence.

**Table 3:** Candidate gut-related mechanisms that could modify bone outcomes under low energy availability.

Pathway	LEA-related trigger	Bone-relevant effect	Level of evidence	Preclinical evidence and context	Athlete-specific status (level 1 and 2)
SCFAs	Lower fermentable substrate intake, altered diet diversity or microbial depletion of SCFA-producing taxa.	Barrier support, immunomodulation, osteoclast suppression and possible IGF-1/Wnt-related effects.	Level 2, 3, 4	Preclinical gut-bone studies; anorexia nervosa and undernutrition analogues; osteoporosis literature.	Not directly tested in LEA-defined athlete cohorts.
Gut barrier and endotoxemia	Energetic stress, high training load, gastrointestinal symptoms or dysbiosis may impair epithelial integrity.	LPS and inflammatory cytokines may increase RANKL-mediated osteoclastogenesis and suppress osteoblast function.	Level 3, 4	General barrier biology, gut-bone and inflammatory bone literature.	Requires athlete studies measuring permeability/inflammation with EA and bone outcomes.
Insulin-osteocalcin crosstalk	LEA can reduce fasting insulin and alter glucose availability.	Osteoblast insulin receptor signalling supports bone acquisition and osteocalcin-mediated energy-bone feedback.	Level 4	Primarily preclinical and metabolic physiology studies.	Promising adjunct marker; causal relevance to REDs remains untested.
Bile acids	Changes in fat intake, microbial bile acid transformation or gut function.	FXR/TGR5 pathways may influence inflammation, osteoclastogenesis and fat-soluble nutrient handling.	Level 4	Emerging gut-bone and metabolic literature.	Speculative for athletes; should be secondary to SCFA/barrier hypotheses.
Amino acid metabolites	Altered protein intake, amino acid oxidation, microbial tryptophan/proline/hydroxyproline metabolism.	May affect collagen substrate supply, AhR signalling, immune tone, barrier function and osteoblast/osteoclast metabolism.	Level 4	Skeletal-cell metabolism, metabolomics and gut microbial amino-acid metabolism studies.	Relevant for metabolomics panels; not established as REDs mechanism.
Prebiotic oligosaccharides	Low fibre or restricted carbohydrate intake may reduce fermentable substrates.	May increase SCFAs and calcium absorption; possible support for microbial resilience.	Level 4	Prebiotic, mineral absorption and animal gut-bone literature.	Potential adjunct only after adequate energy availability is restored.

These pathways are proposed as testable hypotheses rather than established causal mechanisms in athletes.

### 6.1 Short-chain fatty acids

SCFAs are the most mature mechanistic link between gut microbial function and bone. Acetate, propionate and butyrate are produced through microbial fermentation of dietary fibres and other non-digestible substrates (84, 107). Under LEA, SCFA production could be reduced through lower carbohydrate and fibre intake, reduced substrate flow to the colon, altered gut transit, stress-related changes in gut physiology or shifts in microbial community structure. Clinical undernutrition studies reporting lower SCFA-producing taxa and lower SCFA concentrations make this pathway plausible, although direct athlete data are absent (26, 32–34, 59, 62).

SCFAs may influence bone through several routes. They support epithelial barrier integrity, which may reduce endotoxin translocation and systemic inflammation (24, 63, 64). They can regulate immune cell differentiation and inflammatory cytokine production, thereby influencing osteoclastogenic signalling (65–67). In preclinical work, SCFAs have been shown to regulate systemic bone mass and protect against pathological bone loss, in part through effects on osteoclast metabolism and osteoanabolic pathways (22). SCFAs have also been linked to IGF-1 and Wnt-related signalling, providing a bridge between microbial metabolism and established endocrine regulators of bone (21, 22, 68).

For athletes, SCFAs are attractive because they connect diet quality, carbohydrate/fibre availability, gut function and bone remodelling. However, they should not be presented as a proven biomarker of REDs-related bone risk. Future studies should measure stool and/or circulating SCFAs alongside detailed dietary intake, training load, energy availability, endocrine markers and bone outcomes to determine whether SCFA availability explains variance in bone turnover or stress injury risk beyond established REDs indicators.

### 6.2 Gut barrier function, endotoxemia and immune tone

The gut barrier provides a second plausible pathway. Energy deficiency, psychological stress, high training load, gastrointestinal symptoms and altered microbial ecology may compromise epithelial integrity. If barrier function deteriorates, microbial products such as lipopolysaccharide may translocate into circulation, promoting low-grade inflammation. Inflammatory cytokines including tumour necrosis factor-alpha, interleukin-1 beta and interleukin-6 can stimulate osteoclastogenesis through RANKL-mediated pathways and suppress osteoblast function (66, 67, 69, 70, 87, 88, 100).

This pathway is particularly relevant because it could interact with endocrine REDs mechanisms. Low oestrogen or testosterone, low IGF-1, low T3 and elevated cortisol can already shift remodelling towards reduced formation or increased resorption (1, 36, 37). Low-grade inflammation may amplify this shift by increasing osteoclast activity and suppressing osteoblast differentiation. In this sense, gut barrier dysfunction is not a separate mechanism but a potential amplifier of the endocrine and metabolic consequences of LEA.

### 6.3 Insulin, osteocalcin and energy-bone crosstalk

Insulin is relevant because LEA can reduce fasting insulin in athletes and because osteoblasts express insulin receptors that participate in bone acquisition and energy metabolism (71–73). Experimental work in mice indicates that osteoblast-specific insulin receptor signalling supports osteoblast differentiation, bone formation and osteocalcin production (71). Related work suggests that insulin signalling in osteoblasts integrates bone remodelling with glucose homeostasis by influencing osteocalcin activation during bone resorption (72, 73).

The translational relevance to athletes should be stated cautiously. The insulin-osteocalcin loop has been characterised largely in preclinical models and metabolic physiology, not in REDs cohorts. However, it adds an important layer to the LEA-bone model because insulin is both a nutrient-sensitive hormone and a bone-active signal. Under LEA, lower insulin may contribute to reduced osteoblast activity and may interact with low IGF-1, low leptin and altered glucose availability. In a gut-bone framework, microbial metabolites such as SCFAs could influence insulin sensitivity and IGF-1 signalling, whilst energy deficiency could suppress insulin and osteocalcin-mediated crosstalk. This provides a coherent reason to include fasting insulin, glucose and osteocalcin or undercarboxylated osteocalcin in future integrated studies, whilst avoiding strong causal claims.

### 6.4 Bile acids and fat-soluble nutrient handling

Gut bacteria also modify bile acid pools through deconjugation and transformation of primary bile acids into secondary bile acids (115). Bile acids act as signalling molecules through receptors such as farnesoid X receptor (FXR) and Takeda G-protein-coupled receptor 5 (TGR5), which influence energy metabolism, inflammation, and osteoclastogenesis (74, 75). Beyond signalling in bone cells, bile acids regulate cholesterol and lipid homeostasis and influence absorption of fat-soluble nutrients such as vitamins D and K (74, 75, 115). Under LEA, reduced fat intake, altered gastrointestinal motility or microbial shifts could plausibly affect these pathways, but direct athlete evidence is lacking. In athletes with LEA, a combination of reduced fat intake, altered gastrointestinal motility, and microbial shifts could plausibly disrupt these pathways, contributing to both metabolic and skeletal impairments. This mechanism represents an important candidate for future metabolomic and lipidomic profiling in athletic cohorts, though it remains an emerging pathway.

### 6.5 Amino acid metabolites

Amino acid metabolism is a broader and potentially relevant pathway through which the gut microbiome may influence skeletal health. Skeletal cells require amino acids for collagen synthesis, matrix production, redox regulation and osteoblast and osteoclast function (76). Metabolomic studies link amino acid-related pathways with bone turnover markers and low BMD, although causal direction is often unclear (77, 78). Recent LEA metabolomics work suggests that short-term energy deficiency can alter circulating amino acids and related metabolic pathways, supporting the idea that amino acid availability and utilisation shift during under-fuelling (79).

Tryptophan metabolism remains important because microbial indole derivatives and host kynurenine pathway metabolites can influence aryl hydrocarbon receptor (AhR) signalling, mucosal immunity, barrier function and inflammatory tone (67, 75, 77). However, additional amino acid pathways may also matter. Gut microbial metabolism of hydroxyproline, an abundant collagen-derived amino acid, can generate pyrroline-5-carboxylate (P5C), linking microbial activity with proline and glutamate-related metabolism (80). P5C is a metabolic intermediate connecting proline, glutamate and ornithine pathways, and may be relevant to nitrogen handling and microbial cross-feeding. Separately, amino acid signalling pathways, including arginine and tryptophan catabolism, shape immune responses and inflammation (81). These mechanisms are not yet established in athlete bone health, but they justify broadening future metabolomics beyond SCFAs alone.

### 6.6 Oligosaccharides and prebiotic substrates

Prebiotic oligosaccharides, including inulin-type fructans, fructooligosaccharides and galactooligosaccharides, are relevant because they can increase fermentable substrate availability, SCFA production and calcium absorption (82). In gut-bone models, this provides a nutritional link between diet composition, microbial function and mineral metabolism. However, the evidence is stronger in animal models, adolescents, postmenopausal women or general mineral absorption studies than in athletes with REDs. Therefore, oligosaccharides should be discussed as a future intervention candidate rather than as an established treatment. In athletes, any prebiotic strategy would need to consider total energy availability, gastrointestinal tolerance, training timing and whether fibre intake is restricted during heavy training or weight-management periods.

### 6.7 Spaceflight and other catabolic analogues

Spaceflight research offers another mechanistic analogue because microgravity produces rapid skeletal unloading, bone loss and changes in host metabolism. Bedree et al. reported that specific host metabolite and gut microbiome alterations were associated with bone loss during spaceflight in a rodent model (83). This evidence is outside the direct scope of sport nutrition, but it is useful because it shows that gut microbial and metabolite changes can track with bone loss in a catabolic, unloading environment. For athletes, the relevance is conceptual: skeletal outcomes reflect both metabolic/endocrine status and mechanical loading. The spaceflight literature should therefore be cited briefly to support the integrated model, not expanded into a separate review theme.

## 7 Proposed integrated model for athletes

**Figure 1:**
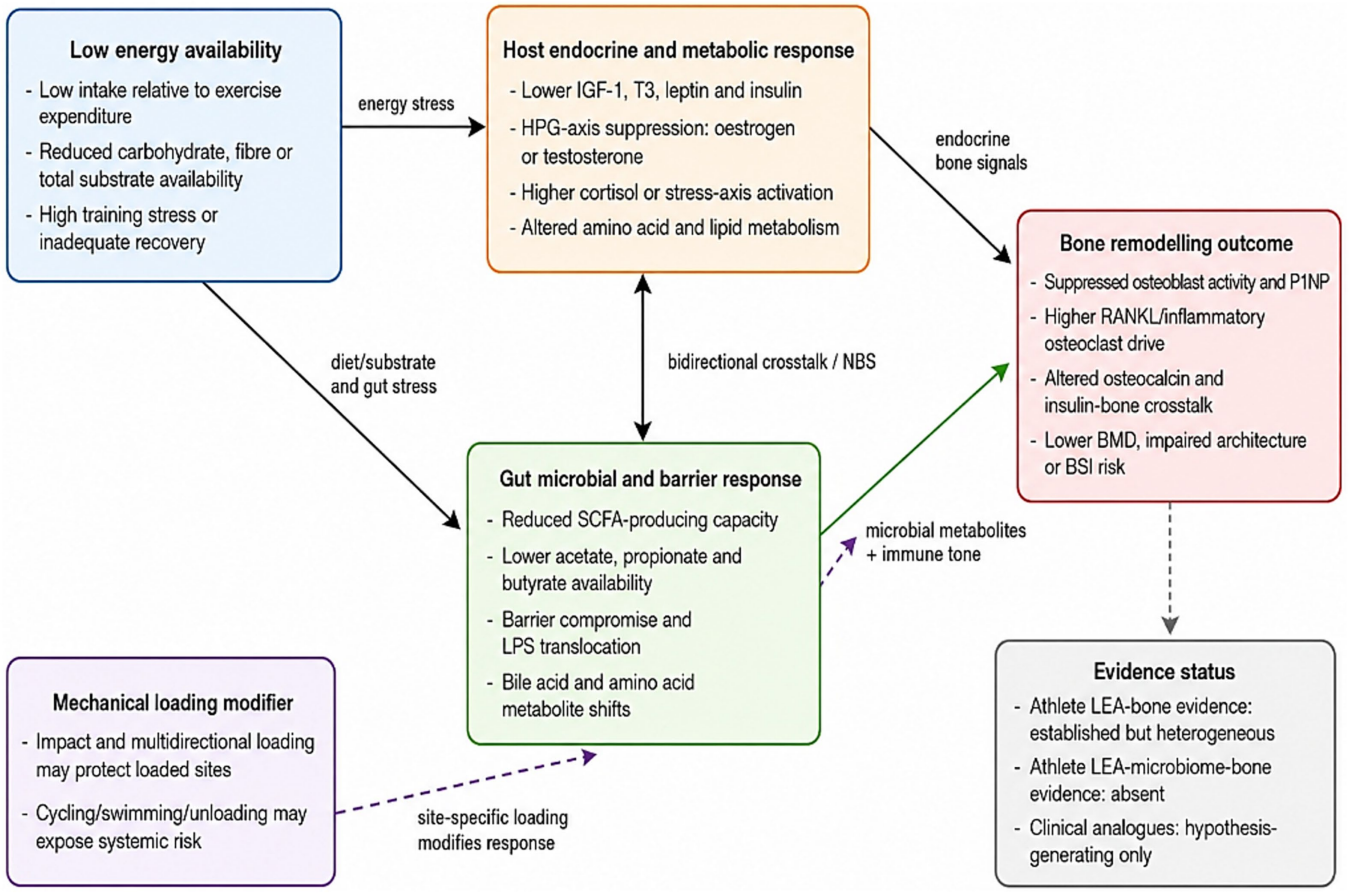
Proposed LEA-gut-bone framework in athletes. Solid arrows show the proposed physiological direction of established or biologically plausible LEA-related endocrine, metabolic, gut microbial and skeletal pathways. The green arrow highlights candidate gut microbial and immune contributions to bone remodelling that remain to be directly demonstrated in athletic cohorts. The purple dotted arrow indicates that mechanical loading may modify the gut-bone response in a site-specific manner, and the grey dotted arrow links the proposed framework to the current evidence-status summary. Mechanical loading is shown as a site-specific modifier because impact and multidirectional loading may partly protect loaded skeletal regions, whereas low-impact or unloading sports may expose systemic energetic risk more clearly. The framework is hypothesis-generating: athlete LEA-bone evidence is established but heterogeneous, whereas direct athlete evidence linking LEA, gut microbiome function and bone outcomes is currently absent. LEA, low energy availability; REDs, relative energy deficiency in sport; SCFA, short-chain fatty acid; LPS, lipopolysaccharide; HPG, hypothalamic–pituitary-gonadal; BSI, bone stress injury.

The proposed integrated model of LEA, the gut microbiome, and bone health in athletes is illustrated in Figure 1. This model summarises the conceptual gut-bone framework and highlights the distinction between established Level 1 athlete LEA-bone evidence, hypothesis-generating Level 3 clinical analogues, and the currently absent direct athlete LEA-microbiome-bone evidence. The pathway begins with established REDs physiology: LEA reduces the energy available for non-exercise physiological functions and can suppress reproductive hormones, IGF-1, T3, leptin and insulin whilst increasing cortisol or other stress-related signals (1, 36, 37). These changes reduce osteoblast activity, impair bone formation and may increase resorption, particularly when combined with low sex steroid exposure or high training stress.

The gut microbiome may modify this pathway through substrate-sensitive microbial metabolism. Reduced energy intake, restricted carbohydrate intake, low fibre intake or altered dietary diversity may reduce fermentable substrates and shift microbial composition. If SCFA-producing capacity falls, barrier integrity and anti-inflammatory signalling may weaken. If gut permeability and endotoxin exposure increase, inflammatory cytokines may stimulate osteoclastogenesis and further suppress osteoblast function. At the same time, LEA-related endocrine changes may feed back on gut function by altering motility, epithelial renewal, immune tone and stress physiology. The result may be a reinforcing loop in which under-fuelling affects both host endocrine systems and microbial function, and both systems converge on bone remodelling.

A further dimension is the bidirectional interaction between psychological stress, appetite regulation and gastrointestinal function. Chronic energy deficiency can be accompanied by mood disturbance, anxiety and maladaptive eating or exercise behaviours (2). Because these factors may also affect gut motility, dietary intake and barrier function, future studies should assess psychological symptoms alongside microbial and endocrine outcomes. This pathway remains hypothetical in athletes. The gut microbiome plays a key role in synthesising and regulating neurotransmitters (e.g., serotonin, gamma-aminobutyric acid), short-chain fatty acids, and immune signals that communicate with the central nervous system via the vagus nerve and systemic circulation. Under LEA, microbial dysbiosis and compromised barrier function may exacerbate neuroinflammatory pathways and disrupt appetite-regulating hormones (e.g., peptide YY, ghrelin), potentially reinforcing maladaptive eating behaviours and psychological stress. Conversely, central nervous system stress can alter gut motility, mucosal barrier function, and secretion, creating a feedback loop that exacerbates microbial and metabolic dysfunction. Whilst the clinical relevance of this bidirectional pathway remains to be established in athletic cohorts, it represents an important integrative component of the wider LEA-gut-bone framework.

Mechanical loading is a key modifier. In impact and multidirectional sports, local osteogenic stimuli may partially offset systemic energetic stress. In cycling, swimming or other low-impact endurance sports, the skeleton may receive less protective loading, making metabolic and endocrine stress more visible at the hip and spine. This model explains why some findings appear anomalous when interpreted only through calculated energy availability: the final bone phenotype reflects the history of energy status, endocrine adaptation, gut-derived signals, mechanical loading and measurement timing.

The model should be treated as testable rather than proven. The most direct hypothesis is that athletes with LEA or higher REDs risk will show lower SCFA availability, altered gut barrier or inflammatory markers and suppressed bone formation compared with energy-replete athletes matched for sport, sex and loading exposure. A second hypothesis is that microbial functional markers will explain additional variance in P1NP, beta-CTX, BMD, microarchitecture or bone stress injury beyond traditional REDs markers. A third hypothesis is that restoration of energy availability, adequate carbohydrate and fibre intake, and appropriate loading will improve microbial function and bone turnover over different timescales.

## 8 Future research priorities

Future studies should move from broad association to integrated, athlete-centred designs. The first priority is prospective cohort work in athletes at risk of REDs, especially endurance, aesthetic, weight-category and low-impact sports. Studies should report the method used to estimate energy availability, the severity and duration of LEA, REDs CAT2 or comparable clinical risk indicators, training load, sport type and mechanical loading characteristics. Because current energy availability is difficult to measure, studies should combine dietary records, exercise expenditure, body composition, menstrual or reproductive status, resting metabolic rate or RMR ratio, endocrine markers and clinical history rather than rely on a single calculation (2, 48).

The second priority is microbial functional assessment. Composition alone is unlikely to be sufficient. Studies should include alpha and beta diversity, taxonomic profiles, SCFA-producing taxa, stool and/or circulating SCFAs, and ideally shotgun metagenomics or metabolomics to capture functional pathways. Sampling should be standardised relative to recent training, diet, menstrual phase where relevant, antibiotic use, probiotic or prebiotic use, gastrointestinal symptoms and travel (99). Dietary assessment should include not only energy intake but also carbohydrate availability, fibre, resistant starch, oligosaccharide intake, protein amount and distribution, fat intake and calcium and vitamin D status.

The third priority is bone phenotyping. DXA-derived BMD remains clinically useful, but it should be complemented where possible by pQCT or HR-pQCT measures of geometry, cortical and trabecular structure, and strength indices. Bone turnover markers such as P1NP and beta-CTX should be collected under standardised fasting and timing conditions, and interpreted alongside menstrual status, sex hormones, IGF-1, T3, cortisol, leptin, insulin and glucose. Prospective bone stress injury surveillance would provide the most clinically meaningful endpoint.

Finally, intervention studies should be staged carefully. The first intervention for REDs remains restoration of adequate energy availability and appropriate clinical management. We emphasise that there is currently insufficient evidence to recommend gut-directed interventions as primary or standalone treatments for REDs-related bone impairment in athletes. Any gut-directed strategies, such as increasing fermentable carbohydrate, prebiotic oligosaccharides, or probiotic supplementation, must be treated as experimental and strictly adjunctive to the primary, non-negotiable clinical requirement: the restoration of adequate energy availability. They should not be positioned as substitutes for energy restoration. Their potential role is adjunctive: once energy balance is restored, prebiotic substrates or specific probiotics might help accelerate microbial recovery, support barrier integrity and facilitate calcium absorption, thereby helping to optimise skeletal recovery.

## 9 Conclusion

LEA is a clinically important contributor to impaired skeletal health in athletes, but the variability in bone outcomes cannot be explained by energy availability alone. The gut microbiome is a biologically plausible modifier because microbial metabolites, barrier function, immune tone and endocrine crosstalk intersect with known REDs pathways. Evidence from athletes supports the LEA-bone relationship, whilst evidence from clinical undernutrition, osteoporosis, preclinical and spaceflight models supports candidate mechanisms involving SCFAs, inflammation, insulin-osteocalcin signalling, bile acids and amino acid metabolites. Direct athlete evidence linking LEA, gut microbiome function and bone outcomes is still absent. The main contribution of the current framework is therefore to define testable hypotheses and practical measurement priorities for future REDs research, whilst maintaining a clear distinction between athlete evidence and clinical analogues.
