# Supplementary material for: Low energy availability, the gut microbiome, and bone health in athletes: a mechanistic narrative review based on athlete evidence and clinical analogues
Source: Front Nutr. 2026 Jul 20;13:1901299. doi: 10.3389/fnut.2026.1901299 (PMC13429644; doi:10.3389/fnut.2026.1901299)
Supplement: Supplementary file 1 [file Table_1.DOCX]

# Low Energy Availability, the Gut Microbiome, and Bone Health in Athletes: A Mechanistic Narrative Review Based on Athlete Evidence and Clinical Analogues

**Supplementary Material: Supplementary Tables S1–S4**

**Table S1.** Detailed summary of studies examining the relationship between energy availability (EA) and bone mineral density (BMD) in athletic and physically active populations. Abbreviations: BMD, bone mineral density; BSI, bone stress injury; CAT1, Clinical Assessment Tool version 1; CAT2, Clinical Assessment Tool version 2; DXA, dual-energy X-ray absorptiometry; EA, energy availability; EDE-Q, Eating Disorder Examination Questionnaire; LEA, low energy availability; LEAF-Q, Low Energy Availability in Females Questionnaire; MD, menstrual dysfunction; P1NP, procollagen type I N-terminal propeptide; RED-S, Relative Energy Deficiency in Sport; RMR, resting metabolic rate; SEAQ-I, Sport Specific Energy Availability Questionnaire - Interview.

| Reference | Study Design & Population | Methodology (EA Assessment) | Key Findings |
| --- | --- | --- | --- |
| Ahmad et al. (2018) | Cross-sectional. Females, n=85. | EA (Dietary Records) | No significant association between low-BMD and calculated EA. |
| Barrack et al. (2023) | Cross-sectional. Adolescent female athletes, n=464. | MD, EDE-Q | ↓ Total body BMD and Lumbar spine BMD in athletes with LEA indicators. |
| Chen et al. (2021) | Cross-sectional. Male runners vs cyclists, n=37. | EA (Dietary Records) | In Cyclists: Lowest EA tertile had ↑ femoral neck/hip BMD. Highest EA tertile had lower bone values. |
| De Souza et al. (2008) | Observational (2-3 mo). Exercising women, n=44. | EA (Dietary Records), Estrogen | ↓ Lumbar spine BMD in Estrogen Deficient groups. |
| Gimunová et al. (2024) | Cross-sectional. Recreational female athletes, n=24. | LEAF-Q | No significant association between low-BMD and LEA risk. |
| Haines et al. (2023) | Cross-sectional. Male runners (n=20) vs Controls. | EA (Dietary Records) | ↓ Lumbar spine BMD in runners with EA <median. ↑ Hip BMD in runners with EA ≥median. |
| Heikura et al. (2017) | Cross-sectional. Runners/walkers, n=59. | EA, MD, Testosterone | ↓ Total body BMD in Amenorrheic vs Eumenorrheic females. No significant difference in males based on EA alone. |
| Heikura et al. (2023) | Cross-sectional. Elite athletes, n=213. | REDs CAT2 | Graded decline in Lumbar Spine & Femoral Neck BMD across risk categories (Yellow, Orange, Red). |
| Ikegami et al. (2022) | Cross-sectional. Pubescent female athletes, n=21. | EA (Dietary Records) | No significant correlation between LEA and BMD. |
| Kalpana et al. (2022) | Cross-sectional. Male Kho-Kho players, n=52. | EA, REDs CAT1 | ↓ Z-score total body BMD in LEA group (EA ≤ 25). |
| Keay et al. (2018) | Cross-sectional. Male cyclists, n=50. | SEAQ-I | ↓ Lumbar spine and Femoral neck BMD in LEA group. |
| Keay et al. (2019) | RCT (6 months). Male cyclists, n=45. | SEAQ-I | ↑ Lumbar spine BMD in the intervention group (improved nutrition/loading). |
| Kyte et al. (2023) | Cross-sectional. Elite female runners vs controls, n=30. | LEAF-Q | Significant negative correlation between LEAF-Q score (LEA risk) and BMD in runners. |
| Lane et al. (2021) | Cross-sectional. Male endurance athletes, n=60. | EA (Dietary Records) | ↑ Total Body BMD in LEA group. |
| Lee et al. (2020) | Observational (1 mo). Male soccer players, n=12. | EA (Dietary Records) | No significant difference in BMD between EA groups. |
| Melin et al. (2015) | Cross-sectional. Female endurance athletes, n=40. | EA, MD | No direct association with MD or EA in this cohort. |
| Meng et al. (2020) | Cross-sectional. Aesthetic sports athletes, n=166. | LEAF-Q, EDI-3 | ↓ Total body, Arms, Legs BMD in the LEA-risk subgroup. |
| Moore et al. (2021) | Cross-sectional. Male endurance athletes, n=14. | EA (Dietary Records) | No significant difference in BMD between groups. |
| Rogers et al. (2021) | Cross-sectional. Female athletes, n=75. | LEAF-Q | Correlation between "Injury" and "Menstrual function" sections of LEAF-Q with low BMD. |
| Stangerup et al. (2025) | Cross-sectional. Elite female athletes, n=19. | MD (Amenorrhea) | ↓ Whole Body, Lumbar Spine, and Proximal Femur BMD in Amenorrheic group compared to Eumenorrheic. |
| Wyatt et al. (2023) | Cross-sectional. Winter endurance athletes, n=44. | REDs CAT1 | ↓ Femoral neck BMD and ↓ Cortical bone area/strength in participants with risk of REDs. |

**Table S2.** Detailed summary of studies examining the effects of energy availability (EA) on biochemical markers of bone metabolism. Abbreviations: β-CTX, beta-isomer of C-terminal telopeptide of type I collagen; BMD, bone mineral density; BSAP, bone-specific alkaline phosphatase; DXA, dual-energy X-ray absorptiometry; EA, energy availability; LEA, low energy availability; LEAF-Q, Low Energy Availability in Females Questionnaire; LH, luteinizing hormone; MD, menstrual dysfunction; P1NP, procollagen type I N-terminal propeptide; PICP, procollagen type I C-terminal propeptide; PINP, procollagen type I N-terminal propeptide; RED-S, Relative Energy Deficiency in Sport; RMR, resting metabolic rate; TRACP-5b, tartrate-resistant acid phosphatase 5b.

| Reference | Study Design & Population | Methodology (EA Assessment) | Key Findings |
| --- | --- | --- | --- |
| Clayton et al. (2020) | RCT (24h severe restriction). Active M+F, n=16. | EA (Dietary Records) | No significant changes in PTH, β-CTX, or P1NP after only 24 hours. |
| De Souza et al. (2008) | Observational (2-3 mo). Exercising women, n=44. | EA, Estrogen | EnD + E2D: ↓ P1NP, ↑ U-CTX-I.   EnD only: ↓ Osteocalcin. |
| Hutson et al. (2023) | RCT (3 days). Females, n=19. | EA (Dietary Records) | LEA group: ↓ P1NP, ↑ β-CTX. |
| Ihle & Loucks (2004) | RCT (5 days). Young women, n=29. | EA (Dietary Records) | Dose-dependent ↓ Osteocalcin & ↓ PICP (at 10, 20, 30 kcal/kgLBM). ↑ NTX at 10 kcal level. |
| Kyte et al. (2023) | Cross-sectional. Elite female runners, n=30. | LEAF-Q | No significant associations between LEA markers and bone turnover markers. |
| Lane et al. (2021) | Cross-sectional. Male endurance athletes, n=60. | EA (Dietary Records) | No significant difference in ALP. |
| Lee et al. (2020) | Observational (1 mo). Male soccer players, n=12. | EA (Dietary Records) | No significant difference in bone turnover markers between groups. |
| McGuire et al. (2024) | Cross-sectional. Male endurance athletes, n=13. | EA (Dietary Records) | In LEA group: ↓ P1NP and ↑ β-CTX. |
| Papageorgiou et al. (2017) | RCT (9 days). Active Females (n=11) & Males (n=11). | EA (Dietary Records) | Women (LEA): ↓ P1NP, ↑ β-CTX.   Men: No significant changes. |
| Papageorgiou et al. (2018) | RCT (3 days). Female recreational athletes, n=10. | EA (Dietary Records) | LEA by nutrition (vs. exercise) caused ↓ P1NP. |
| Sim et al. (2024) | RCT (4 days). Male endurance runners, n=12. | EA (Dietary Records) | No significant changes in Osteocalcin, β-CTX, or P1NP. |
| Stangerup et al. (2025) | Cross-sectional. Elite female athletes, n=19. | MD (Amenorrhea) | No significant difference between groups in P1NP or CTX. |
| Zanker et al. (1998) | Cross-sectional. Females, n=27. | MD (Amenorrhea) | Amenorrheic runners: ↓ Osteocalcin, ↓ BAP, ↓ PICP, ↓ Pyr, ↓ Dpyr. |

**Table S3.** Detailed summary of studies examining gut microbiota alterations in anorexia nervosa and related undernutrition states. Abbreviations: AN, anorexia nervosa; AN-BP, anorexia nervosa binge-purging subtype; AN-R, anorexia nervosa restricting subtype; AN-REC, anorexia nervosa weight-restored/recovering; BN, bulimia nervosa; BED, binge eating disorder; CR, caloric restriction; ED, eating disorder; FMT, fecal microbiota transplant; HC, healthy controls; RDI, recommended daily intake; SCFA, short-chain fatty acid; VLCD, very low calorie diet.

| Reference | Study Design | Population | Methodology | Key Microbial Findings |
| --- | --- | --- | --- | --- |
| Andreani et al. (2023) | Observational longitudinal | Females (n=91): 57 AN vs 34 HC. | 16S rRNA | Diversity: Longitudinal ↓ α-diversity in AN vs HC.   Taxa: ↓ *Dialister*, *Legionella*.   Follow-up: ↑ *Escherichia-Shigella*, *Alistipes*. |
| Armougom et al. (2009) | Observational | AN (n=9) vs Obese (n=20) vs HC (n=20). | 16S rRNA | Taxa: ↑ *Methanobrevibacter smithii*in AN (energy harvest adaptation). |
| Borgo et al. (2017) | Observational | Females (n=30): 15 AN vs 15 HC. | 16S rRNA | Diversity: Significant β-diversity shift in AN.   Taxa: ↑ *Enterobacteriaceae*.   ↓ *Roseburia*, *Clostridium*, *Ruminococcus*.   Metabolites: ↓ Total SCFAs, ↓ Butyrate, ↓ Propionate. |
| Castellini et al. (2023) | Cross-sectional | Females (n=75): AN vs BN vs BED vs HC. | 16S rRNA | Diversity: ↓ α-diversity in all ED groups.   Taxa: ↓ Butyrate-producers (e.g., *Roseburia*, *Anaerostipes*) in EDs.   ↑ *Bacteroides* in AN.   Metabolites: ↓ Butyric/Propionic acid in EDs. |
| Cattaruzza et al. (2022) | Cross-sectional | Females (n=327): AN vs AN-REC vs HC. | / | Taxa: ↓ *Roseburia* in active AN (suggests loss of SCFA production capacity). |
| De La Torre-Luque et al. (2022) | Observational longitudinal | Females (n=41): 22 AN vs 19 HC. | 16S rRNA | Taxa: ↑ *Blautia*, *Lachnospiraceae* in AN at discharge.   Weight gain increased *Faecalibacterium*, *Dialister*.   ↓ *Bacteroides*, *Anaerostipes* during refeeding. |
| Fan et al. (2023) | Human + Animal Model | AN patients + Germ-free mice (FMT). | Metagenomics | Function: FMT from AN patients caused impaired weight gain and ↑ gut permeability in mice.   Metabolites: ↓ Butyrate and Propionate in AN donors. |
| Fouladi et al. (2022) | Longitudinal | Total n=191 (93 AN, 98 HC). | Metagenomics | Diversity: ↓ Shannon index (metabolic pathways).   Taxa: ↓ *Faecalibacterium* prausnitzii, *Bifidobacterium adolescentis*. |
| Gong et al. (2024) | Intervention (VLCD) | n=14 (Obese/DM2): Very Low Calorie Diet. | 16S rRNA | Taxa: ↑ Bacteroidetes.   ↓ *Firmicutes*/Bacteroidetes ratio, *Escherichia-Shigella*. |
| Hanachi et al. (2019) | Case-control | Females (n=55): 33 AN vs 22 HC. | 16S rRNA | Diversity: ↓ α-diversity in AN.   Taxa: ↑ Pathobionts (*Klebsiella*, *Salmonella*).   ↓ *Roseburia*, *Anaerostipes*. |
| Käver et al. (2024) | Longitudinal | Females (n=104): 63 AN vs 41 HC. | 16S rRNA | Taxa: ↓ *Ruminococcus*, *Anaerostipes* in AN.   Recovery showed partial normalization. |
| Kleiman et al. (2015) | Longitudinal | Females (n=28): 16 AN vs 12 HC. | 16S rRNA | Diversity: ↓ α-diversity in AN.   Taxa: ↓ *Anaerostipes*, *Faecalibacterium* before treatment. |
| Mack et al. (2016) | Observational | Females (n=110): 55 AN vs 55 HC. | 16S rRNA | Diversity: ↑ Diversity after weight gain.   Taxa: ↑ *Methanobrevibacter*, *Firmicutes*.   ↓ Bacteroidetes, *Roseburia*. |
| Mondot et al. (2022) | Retrospective | Females (n=164): 90 AN vs 74 HC. | 16S rRNA | Taxa: Consistent reduction of *Roseburia* in AN. |
| Monteleone et al. (2020) | Longitudinal | Females (n=41): AN Admission vs Discharge vs HC. | 16S rRNA | Diversity: ↓ α-diversity in acute AN; ↑ after weight restoration.   Taxa: Acute AN: ↑ *Actinobacteria*. |
| Monteleone et al. (2021) | Observational | Females (n=43): AN-R vs AN-BP vs HC. | 16S rRNA | Diversity: ↓ α-diversity in AN.   Taxa: ↑ *Bifidobacterium* in AN-BP.   Metabolites: ↓ SCFAs (Acetate, Propionate, Butyrate). |
| Morita et al. (2015) | Cross-sectional | Females (n=46): 25 AN vs 21 HC. | 16S rRNA | Diversity: Significant β-diversity shift.   Taxa: ↓ *Bacteroides*, *Clostridium*clusters (IV, XIVa).   ↑ *Clostridium* cluster XVIII. |
| Mörkl et al. (2017) | Cross-sectional | Females (n=106): AN vs HC vs Athletes. | 16S rRNA | Diversity: ↓ α-diversity in AN/Obese vs Athletes.   Taxa: ↑ *Coriobacteriaceae* in AN. |
| Prochazkova et al. (2021) | Observational | Females (n=126): 59 AN vs 67 HC. | 16S rRNA | Taxa: ↑ *Alistipes*, *Christensenellaceae*.   ↓ *Faecalibacterium*, *Bacteroides*.   Metabolites: ↓ Acetate, Butyrate, Propionate. |
| Schulz et al. (2021) | Longitudinal | Adolescent females: AN vs HC. | 16S rRNA | Diversity: Gut microbiota alteration in AN does not normalize with short-term weight restoration. |
| Yuan et al. (2022) | Observational | Total n=60 (30 AN, 30 HC). | 16S rRNA | Diversity: Significant β-diversity shift.   Taxa: ↑ *Lachnospiraceae*.   ↓ *Faecalibacterium*, *Ruminococcaceae*. |
| Zou et al. (2020) | Intervention (CR) | n=41: Caloric Restriction (60% RDI). | Metagenomics | Taxa: Shifts in *Prevotella* vs *Bacteroides*.   Associations between *Clostridium*and BMI loss. |

**Table S4.** Detailed summary of studies examining the relationship between gut microbiota, fecal short-chain fatty acids (SCFAs), and bone mineral indices in clinical, aged, and general populations. Abbreviations: AhR, aryl hydrocarbon receptor; BMD, bone mineral density; CON/HC, healthy controls; DAO, diamine oxidase; FMT, fecal microbiota transplant; LPS, lipopolysaccharide; OP, osteoporosis; OPN, osteopenia; PMO, postmenopausal osteoporosis; PMW, postmenopausal women; SCFA, short-chain fatty acid; TRACP-5b, tartrate-resistant acid phosphatase 5b; ucOC, undercarboxylated osteocalcin.

| Reference | Study Design | Population | Methodology | Key Microbial Findings & Mechanism |
| --- | --- | --- | --- | --- |
| Chen et al., 2021 | Cross-sectional | Children, n=236 (6-9 years). Low vs Med vs High BMD. | 16S rRNA | Diversity: No significant differences. Taxa: ↑ In Low BMD: *Lachnoclostridium*, *Blautia* Mechanism: Early dysbiosis markers linked to bone mass in childhood. |
| Chen et al., 2024 | Case-control | Females, n=99 (55 OP, 44 HC). | Shotgun | Diversity: ↓ α-diversity in OP. Taxa: ↑ In OP: *Anaerostipes* (genus), *Methanobrevibacter smithii*, *Bifidobacterium animalis*, *Lactobacillus plantarum*, *Carnobacterium mobile*, *Rhodococcus defluvii* ↑ In Controls: *Bacillus luciferensis*, *Acetivibrio cellulolyticus*, *Citrobacter amalonaticus*, *Bifidobacterium breve* Mechanism: Red meat intake associated with OP-enriched taxa. |
| Cheng et al., 2022 | Observational | PMW, n=12 with severe OP (Vit D3+Ca). | 16S rRNA | Diversity: ↑ α-diversity in Severe OP. Taxa: ↑ In Severe OP: *Firmicutes*, *Ruminococcaceae* UCG-014, *Faecalibacterium* ↓ In Severe OP: *Bifidobacterium*, *Actinobacteria*, *Bifidobacterium pseudocatenulatum*, *Lactobacillus* (tendency) Mechanism: Treatment enriched *Actinobacteria* and improved calcium absorption. |
| Das et al., 2019 | Observational | Elderly, n=181 (Normal/OPN/OP). | 16S rRNA | Diversity: no significant differences. Taxa: ↑ In OPN vs OP: *Escherichia/Shigella*, *Veillonella* ↑ In OP vs Normal: Actinomyces, Eggerthella, *Clostridium Cluster XIVa*, *Lactobacillus* Mechanism: Expansion of pro-inflammatory taxa (e.g., Eggerthella). |
| Gao et al., 2024 | Observational | Adults, n=605 (Normal/OPN/OP). | Shotgun | Diversity: no significant differences. Taxa: ↑ In OP Group: *Clostridium leptum*, *Fusicatenibacter saccharivorans*, *Roseburia hominis* ↓ In Low-Genetic-Risk: *Roseburia faecis* ↑ In High-Genetic-Risk: *Turicimonas muris* ↑ In Normal BMD: *Megasphaera elsdenii* ↑ In Females: *Enterobacter bugandensis*, *Intestinimonas butyriciproducens*, *Bifidobacterium longum* ↓ In Males: *Enterobacter mori*, *Bilophila wadsworthia*, *Bacteroides intestinalis* |
| Grahnemo et al., 2024 | Prospective | Adults, n=7043 (Fracture vs No). | Shotgun | Diversity: Positive correlation (PN). Taxa: ↑ In Fracture Cases: *Proteobacteria*, *Gammaproteobacteria*, *Parabacteroides*, *Lachnoclostridium*, *Dorea longicatena* ↑ In Low Fracture Risk: *Tenericutes* Mechanism: LPS biosynthesis (*Proteobacteria*) vs. Amino acid metabolism (*Tenericutes*). |
| Greenbaum et al., 2022 | Observational | Females, n=499 (Post/Perimenopausal). | Shotgun | Taxa: ↓ Negatively associated with BMD:Bacteroidetes (*Bacteroides* vulgatus, B. uniformis, B. fragilis, B. massiliensis), *Fusobacteria* (*Fusobacterium ulcerans*) ↑ Positively associated with BMD:*Firmicutes* (*Clostridium leptum*, *Ruminococcus lactaris*) Mechanism: B. vulgatus negatively regulated BMD via serum valeric acid. |
| He et al., 2020 | Cross-sectional | PMW, n=106 (53 OP, 53 HC). | 16S rRNA | Diversity: ↓ α-diversity in OP. Taxa: ↑ In OPN: *Klebsiella*, Morganella, *Escherichia/Shigella*, Enterobacter, Citrobacter, Pseudomonas, Succinivibrio, Desulfovibrio ↓ In OPN: *Blautia*, Fusicatenibacter, *Lachnospiraceae* ↑ In OP: *Lactobacillales*, *Coriobacteriales*, *Parabacteroides*, *Lactobacillus* ↓ In OP & OPN: *Bacteroides massiliensis*, *Lachnospira pectinoschiza*, *Blautia* Mechanism: Perturbed metabolite profile linked to dysbiosis. |
| Huang et al., 2023 | Observational | Females, n=58 (21 PMO, 37 HC). | 16S rRNA | Diversity: no significant differences. Taxa: ↑ In PMO: *Fusobacteria*, Lactobacillaceae, *Lactobacillus* salivarius, Bacilli, Erysipelotrichia ↓ In PMO: *Ruminococcaceae*, *Bacteroides* eggerthii |
| Keshavarz Azizi (2021) | Pilot study | PMW, n=36 (12 OP, 12 OPN, 12 HC). | qPCR | Taxa: *Akkermansia* muciniphila levels did not differ significantly between groups. |
| Kuo et al., 2023 | Observational | Females, n=47 (OP vs OPN vs HC). | 16S rRNA | Diversity: ↓ α-diversity in OP (NS). Taxa: ↑ In OP: *Dorea*, Erysipelotrichacea, Streptococcus, *Collinsella*, Flavonifactor, Butyricicoccus, Paraprevotella, *Parabacteroides*, Colidextribacter, *Barnesiella*, *Subdoligranulum*, Tyzzerella, *Oscillibacter* ↑ In OPN: *Prevotella*, Eubacterium_rumina, *Blautia*, Alloprevotella, *NK4A214_group* ↑ In OP vs OPN: *Subdoligranulum*, *Collinsella*, *Flavonifractor* Mechanism: Functional metabolic shifts linked to bone loss. |
| Lai et al., 2024 | Observational | Youths, n=62 (Normal vs OPN vs OP). | 16S rRNA | Diversity: ↑ α-diversity in Healthy vs OP. Taxa: ↑ In Healthy BMD: *Bacteroides* ↑ In OPN: *Prevotella* (P. copri), *Megamonas*, Kocuria, Desulfovibrio ↑ In OP: *Faecalibacterium*, *Subdoligranulum*, *Ruminococcus*, Butyricimonas, Saccharimonadaceae ↓ In OPN: *Ruminococcus* bicirculans |
| Li et al., 2019 | Cross-sectional | Adults, n=102 (Low-BMD vs Control). | 16S rRNA | Diversity: no significant differences. Taxa: ↑ In Low-BMD: Bacteroidetes, Bacteroidaceae, *Bacteroides* ↓ In Low-BMD: *Lachnospiraceae*, *Roseburia* ↑ In Controls: *Firmicutes* ↑ Associated with BMD: *Bifidobacterium*, *Lactobacillus*, *Roseburia* Mechanism: Increased LPS in Low-BMD group. |
| Li et al., 2024 (A) | Retrospective | Adults, n=126 (67 OPN vs 59 CON). | 16S rRNA | Diversity: ↓ α-diversity in OPN. Taxa: ↑ In OP: *Escherichia-Shigella*, *Faecalibacterium* prausnitzii, *Enterobacteriaceae* ↓ In OP: *Prevotella* (P. copri), *Megamonas*, Bacteroidales, Rikenellaceae, Tannerellaceae, *Dialister* invisus Mechanism: Decreased starch/sucrose metabolism related to SCFA production. |
| Li et al., 2024 (B) | Retrospective | Females, n=18 (10 PMO, 8 HC). | 16S rRNA | Diversity: Significant β-diversity shift. Taxa: ↓ In PMO: *Subdoligranulum*, Muribaculaceae, *Alistipes* Mechanism: Reduced SCFAs in PMO group. |
| Li et al., 2025 | Prospective | Adults, n=2476 (Rotterdam & Framingham). | 16S rRNA | No significant results related to bone health in this large cohort. |
| Liang et al., 2023 | Cross-sectional | Females, n=26 (Low BMD vs OP vs HC). | 16S rRNA | Diversity: ↓ α-diversity in PMO. Taxa: ↑ In Low BMD/PMO: *Klebsiella*, *Escherichia-Shigella*, *Actinobacteria*, *Proteobacteria*, *Enterobacteriaceae*, *Bifidobacterium*, *Blautia*, *Subdoligranulum*, *Roseburia* ↓ In Low BMD/PMO: *Lactobacillus*, *Akkermansia*, *Prevotella*, *Alistipes*, Butyricicoccus, Bacteroidetes Mechanism: Altered metabolites (e.g., ↓ Tryptophan, ↑ Indole-3-acetic acid). |
| Ling et al., 2021 | Case-control | Adults, n=1776 (OP vs OPN vs HC). | 16S rRNA | Diversity: Significant β-diversity shift. Taxa: ↑ In OP: Actinobacillus, *Blautia*, *Oscillospira*, *Bacteroides*, *Phascolarctobacterium*, Eggerthella, Rikenellaceae ↓ In OP: *Ruminococcaceae*, *Collinsella*, *Veillonellaceae* |
| Liu et al., 2022 | Observational | OA Patients, n=80 (OP vs Normal). | 16S rRNA | Diversity: no significant differences. Taxa: ↑ Increased in OA-OP: Bacteroidetes, Verrucomicrobiota, *Akkermansia*, *Bacteroides* ↓ Decreased in OA-OP: *Prevotella* copri, *Firmicutes*, Actinobacteriota, *Ruminococcaceae*, *Faecalibacterium*, *Dialister*, *Bifidobacterium* |
| Mei et al., 2022 | Observational | Females, n=91 (HIV+ vs HIV-). | 16S rRNA | Diversity: no significant differences. Taxa: ↑ In Low BMD: *Dorea*, Megasphaera, *Lachnospiraceae*, *Ruminococcus*, Mitsuokella ↓ In Low BMD: *Mollicutes* RF39 |
| Authors | Study Design | Population | Method | Key Microbial Findings & Mechanism |
| Okoro et al., 2023 | Cohort | Men and Women (FHS, MrOS cohorts). | 16S rRNA | Taxa: ↑ In Low BMD: *Akkermansia* municiphila ↓ In Low BMD: *Lachnospiraceae*, *Faecalibacterium* Mechanism: ↑ Histidine, purine, and pyrimidine metabolism in low BMD. |
| Orwoll et al., 2020 | Prospective | Men, n=831 (MrOS). | 16S rRNA | Taxa: ↓ Associated with worse bone: Anaerofilum, Ruminiclostridium 9, *Lactobacillus*, Streptococcus ↑ Associated with worse bone: Methanomassiliicoccus (linked to cortical porosity) ↑ Associated with better bone: Tyzzerella |
| Ozaki et al., 2020 | Observational | Females, n=38. | 16S rRNA | Taxa: ↑ In Low BMD/High TRACP-5b: Rikenellaceae ↓ In Fracture History: *Lachnospiraceae* ↑ In High Vitamin K2: *Bacteroides* ↓ In Older Group: *Lachnospiraceae*, *Blautia* ↑ In High ucOC Group: *Sutterella* |
| Palacios-González (2020) | Observational | Females, n=92 (Low BMD vs Normal). | 16S rRNA | Diversity: no significant differences. Taxa: ↑ In Low BMD: γ-*Proteobacteria*, *Klebsiella*, Erwinia, Lachnospira, Yersinia ↓ In Low BMD: Bilophila, Bacteroidales, Paraprevotella, Odoribacteraceae ↑ In Normal BMD: *Akkermansia*, *Actinobacteria* Mechanism: Increased *Enterobacteriaceae* in Vit D deficiency. |
| Qin et al., 2021 | Observational | Elderly, n=29 (OP vs HC). | Shotgun | Diversity: no significant differences. Taxa: ↑ In OP: Streptococcus (sanguinis, gordonii, mitis), Actinomyces (odontolyticus, graevenitzii), *Escherichia* coli, Pantoea ↑ In Control: *Akkermansia* muciniphila, *Bacteroides* (eggerthii, fragilis, uniformis), Butyricimonas Mechanism: OP linked to ↑ LPS biosynthesis, ↑ Phytate degradation, ↓ SCFA. |
| Raftar et al., 2021 | Observational | Adults, n=36 (OP vs OPN vs HC). | 16S rRNA | Taxa: ↑ In Healthy: *Akkermansia* muciniphila (trend, not significant). |
| Rettedal et al., 2020 | Observational | Females, n=86 (OP vs OPN vs HC). | 16S rRNA | Diversity: Significant beta-diversity shift. Taxa: Healthy vs OP: ↓ Clostridia, Methanobacteriaceae, ↑ *Bacteroides* OP vs Healthy: ↑ Betaproteobacteria, *Bacteroides* stercoris, Adlercreutzia, ↓ Turicibacter, Romboutsia OPN vs Healthy: ↑ *Parabacteroides*, *Roseburia*, ↓ Verrucomicrobia Mechanism: ↑ Cyanoamino acid metabolism in OP/OPN. |
| Roselló-Añón et al., 2023 | Case-control | Elderly, n=50 (Hip Fracture vs Control). | 16S rRNA | Diversity: ↑ α-diversity in Fracture. Taxa: ↑ In Fracture: Bacteroidales, Peptostreptococcales, Bacteroidetes ↓ In Fracture: Lachnospirales, *Firmicutes* |
| Teng et al., 2024 | Observational | Uyghur adults, n=58 (OPN vs Control). | 16S rRNA | Diversity: Significant beta-diversity shift. Taxa: ↑ In OPN: Erysipelotrichia, *Clostridium*_sensu_stricto_1, *Lachnoclostridium*, Acidaminococcus, Fusicatenibacter ↓ In OPN: *Phascolarctobacterium* ↑ In Control: Succinivibrio, Sphingomonas |
| Wang et al., 2021 | Observational | Females, n=361. | Shotgun | Diversity: ↓ diversity with low BMD. Taxa: ↓ Negatively correlated with T-score: Eubacterium, *Escherichia*, *Klebsiella*, *Clostridium*, *Blautia* ↑ Positively correlated with T-score: *Prevotella*, *Parabacteroides*, *Megamonas*, *Akkermansia* |
| Wang et al., 2022 | Observational | Adults, n=57. | 16S rRNA | Diversity: ↑ α-diversity in females. Taxa: ↑ In females (Higher BMD): *Firmicutes*, Clostridiales ↑ In females (OP): Peptostreptococcaceae, *Megamonas* ↑ In males: *Lactobacillales* |
| Wang et al., 2023 | Case-control | Females, n=98 (PMO vs Non-PMO). | 16S rRNA | Diversity: ↓ α-diversity in PMO. Taxa: ↑ In PMO: *Veillonella*, *Parabacteroides*, Harryflintia(Bacteria); *Eurotium*, *Penicillium* (Fungi) ↑ In Non-PMO: *Prevotella*, Enterobacterium(Bacteria); *Pichia*, *Auricularia* (Fungi) |
| Wei et al., 2021 | Case-control | Adults, n=108 (OP vs Control). | 16S rRNA | Diversity: Significant beta-diversity shift. Taxa: ↑ In OP: *Bacteroides*, *Eisenbergiella*, *Clostridium*_XlVa, *Coprococcus*, *Lactobacillus*, Eggerthella, *Parabacteroides* ↓ In OP: *Veillonella*, Raoultella Mechanism: ↑ Phytate degradation, ↓ SCFA in OP. |
| Yan et al., 2024 | Observational | Females, n=122 (OP vs Normal). | 16S rRNA | Diversity: ↑ α-diversity in OP. Taxa: ↑ Increased in OP: *Agathobacter*, *Lactobacillus*, *Oscillibacter*, *Prevotella*ceae ↓ Decreased in OP: *Bacteroides*, *Blautia*, Fusicatenibacter, *Ruminococcus*, *Anaerostipes* |
| Yang et al., 2022 | Cross-sectional | Females, n=132 (OP vs OPN vs HC). | 16S rRNA | Diversity: ↑ richness in OPN. Taxa: ↑ In OP: *Megamonas*, *Lachnoclostridium*, Fusicatenibacter, *Bifidobacterium* ↓ In OP: *Ruminococcaceae* ↑ In Controls: Romboutsia, *Weissella* Mechanism: ↓ IL-10, ↑ TNF-α in OP. |
| Zhu et al., 2025 | Observational | Females, n=104 (PMO vs Control). | 16S rRNA | Diversity: ↓ α-diversity in PMO. Taxa: ↑ In PMO: *Roseburia*, *Bacteroides* ↓ In PMO: Streptococcus, *Dorea* Mechanism: ↑ LPS, ↑ D-lactic acid, ↑ DAO in PMO. |
